# Novel functions of FoxM1: from molecular mechanisms to cancer therapy

**DOI:** 10.3389/fonc.2013.00030

**Published:** 2013-03-05

**Authors:** Mónica Alvarez-Fernández, René H. Medema

**Affiliations:** ^1^Cell Division and Cancer Group, Spanish National Cancer Research Center (CNIO)Madrid, Spain; ^2^Department of Cell Biology, The Netherlands Cancer InstituteAmsterdam, Netherlands

**Keywords:** FoxM1, DNA damage, senescence, checkpoint, cancer, therapy

## Abstract

FoxM1 is a member of the forkhead family of transcription factors. Since its identification 15 year ago, numerous studies have progressively contributed to our current understanding on FoxM1 functions. Early work showed that FoxM1 regulates the transcriptional program of the G2 phase of the cell cycle, and is essential for proper mitotic progression and genomic stability. Moreover, FoxM1 was found to be overexpressed in many different types of human cancer, suggesting a role of FoxM1 in tumor proliferation. In the past years, a significant number of studies have formally demonstrated the involvement of FoxM1 in different aspects of tumorogenesis, including angiogenesis, invasion, and metastasis. In addition to this, recent studies have placed FoxM1 in DNA damage response and senescence pathways, two pathways relevant to tumor progression and the response to cancer therapies. Here, we review and discuss the molecular mechanisms through which FoxM1 executes these new roles, and the implications for the potential use of FoxM1 as a therapeutic target in cancer.

## Introduction

FoxM1, also known as Trident, MPP2, or HFH-11, belongs to the large family of forkhead transcription factors (Korver et al., 1997b). Forkhead box (Fox) proteins are a superfamily of evolutionarily conserved transcriptional regulators, defined by a common DNA binding domain termed the forkhead box or winged helix domain (Myatt and Lam, 2007). FoxM1 binds promoter regions with a preference for a consensus “TAAACA” recognition sequence, although with lower affinity than other forkhead proteins (Littler et al., 2010). Its expression is restricted to proliferating cells, and excluded from quiescent and terminally differentiated cells (Korver et al., 1997a; Ye et al., 1997). Its expression, both at the mRNA and protein levels, is cell cycle-regulated: it increases at the entry of S-phase, peaks during G2 and M, and is degraded during mitotic exit (Laoukili et al., 2008b; Park et al., 2008). Similarly, its transcriptional activity is tightly regulated throughout the cell cycle by multisite phosphorylation by different kinases (Fu et al., 2008; Laoukili et al., 2008a; Anders et al., 2011), and its counteracting phosphatases (Alvarez-Fernandez et al., 2011), reaching its maximum activity in the G2 phase of the cell cycle. FoxM1 is a critical cell cycle regulator. It controls the expression of genes required for both G1/S and G2/M transition (Laoukili et al., 2005; Wang et al., 2005); and it is essential for mitotic entry and progression, ensuring the maintenance of chromosome stability (Laoukili et al., 2005).

Amplifications of the 12p13 chromosomal band, comprising the FoxM1 gene, have been reported in numerous tumors such as cervical squamous cell carcinomas, breast adenocarcinomas, nasopharyngeal carcinomas, and head and neck squamous cell carcinomas (Singh et al., 2001). FoxM1 is one of the most common genes overexpressed in solid tumors of prostate, lung, bladder, ovary, colon, liver, breast, kidney, stomach, and pancreas (Pilarsky et al., 2004). It is also aberrantly expressed in other cancers, such as basal cell carcinomas (Teh et al., 2002), glioblastomas (Liu et al., 2006), or acute myeloid leukemia (Nakamura et al., 2010). Recently, it has been reported that FoxM1 expression can also be modulated by microRNAs. FoxM1 has been identified as a direct target of miR-134, whose levels are inversely correlated with the invasive potential of some NSCLC cells (Li et al., 2012). Moreover, FoxM1 is also repressed by miR-370, a tumor suppressor miRNA frequently silenced in acute myeloid leukemia (Zhang et al., 2012b). In addition to its positive role on cell proliferation, FoxM1 has been shown to play roles in other cancer-related processes, such as invasion and metastasis (Dai et al., 2007; Wang et al., 2008; Park et al., 2011; Huang et al., 2012). Moreover, its expression levels correlate with poor prognosis and metastasis in different tumors, suggesting the possibility of using FoxM1 as a prognosis and/or diagnosis marker [revised in (Teh, 2012)]. In the past years, it has also become clear that FoxM1 plays important roles in the DNA damage response and senescence pathways. Early tumor lesions show DNA damage and senescence markers, probably due to oncogene activation and unscheduled proliferation. Importantly, oncogene-induced replicative stress and senescence are tumor barriers that tumor cells need to bypass to allow cancer progression. Moreover, most commonly used cancer treatments rely on the induction of DNA damage, leading to apoptosis, and eventually senescence response, in order to prevent tumor cell expansion (Nardella et al., 2011; Lord and Ashworth, 2012). Therefore, it is critical to understand the specific roles that FoxM1 plays in these pathways in order to design better therapeutic approaches. Here, we summarize those findings on FoxM1 function in DNA damage and senescence processes, and its impact on therapeutic strategies against cancer.

## FoxM1 and the DNA damage response

The first evidence of the implication of FoxM1 in DNA damage pathways came from the observation that FoxM1-deficient cells showed increased levels of DNA damage. Mouse embryonic fibroblasts (MEFs) derived from FoxM1 knockout mouse displayed high levels of the γH2AX marker compared to wild-type MEFs (Tan et al., 2007). Those cells also showed an increased number of TUNEL foci, which efficiently end label sites of DNA breaks, suggesting a defect in DNA repair (Tan et al., 2007). High levels of spontaneous γH2AX foci were also detected in osteosarcoma U2OS cells depleted of FoxM1 by RNA interference, and correlated with decreased levels of X-ray repair cross-complementing protein 1 (*XRCC1*) and breast cancer**–**associated gene 2 (*BRCA2*), two genes involved in DNA repair (Tan et al., 2007). Although both genes were proposed as FoxM1 targets, they have not been proven to be the mediators of such increase in spontaneous damage observed in FoxM1-deficient MEFs (Tan et al., 2007). Indeed, knockdown of FoxM1 in breast cancer cells also led to an increase in DNA damage, but did not result in the downregulation of those potential FoxM1 targets, BRCA2, and XRCC1, neither at protein nor mRNA level (Kwok et al., 2010). These results suggest that FoxM1 might regulate the expression of other genes involved in DNA damage repair pathways.

Interestingly, a recent report has shown that FoxM1 null MEFs are hypersensitive to different DNA damaging insults, such as epirubicin (a topoisomerase II inhibitor), or γ-irradiation (IR), suggesting again a role of FoxM1 in DNA damage repair (Monteiro et al., 2012). In the same study, it was demonstrated for the first time that FoxM1 is required for DNA double strand break (DSB) repair by homologous recombination (HR) but dispensable for non-homologous end-joining (NHEJ) repair (Monteiro et al., 2012). In agreement with that, BRIP (BRCA1-associated BACH1 helicase), a protein involved in HR DSB repair, was identified as a direct transcriptional target of FoxM1. Importantly, ectopic BRIP expression can partially rescue the increased damage and repair deficiency of FoxM1 null cells (Monteiro et al., 2012). This indicates that FoxM1 mediates HR repair at least in part through transcriptional regulation of BRIP, although other targets are likely to be involved in this function of FoxM1. For instance, Rad51, another critical protein required for efficient HR repair, contains 2 forkhead-binding sites in its promoter, and has also been described as a transcriptional target of FoxM1 in glioblastoma cells (Zhang et al., 2012a).

**Figure 1:**
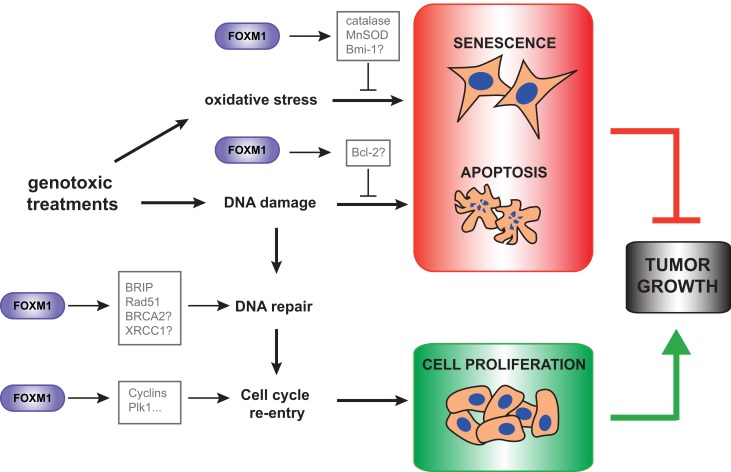
**FoxM1 functions in response to DNA damage and oxidative stress.** FoxM1 regulates the response to DNA damage at different levels. Upon genotoxic treatments, FoxM1 is required for efficient DNA repair and cell cycle resumption once the damage is repaired. Moreover, FoxM1 prevents DNA damage-induced apoptosis, and protect cells from oxidative stress-induced senescence. These FoxM1 functions favor tumor growth, and open different possibilities of targeting FoxM1 in cancer therapies.

FoxM1 has been reported to modulate drug sensitivity and resistance in various tumor types. In breast cancer cell lines with acquired resistance for cisplatin or epirubicin FoxM1 was found to be overexpressed and its depletion was able to re-sensitize these cell lines to the respective genotoxic drug (Kwok et al., 2010; Monteiro et al., 2012). Silencing of FoxM1 also led to higher sensitivity to doxorubicin in breast cancer cells in a xenograft mouse model (Park et al., 2012). FoxM1 has also been linked to genotoxic drug resistance in glioblastoma multiforme (GBM). FoxM1 was found significantly upregulated in recurrent GBM tumor samples compared with primary tumors, and its expression levels correlated with poor response to the alkylator temolozide. In both tumor types, FoxM1-dependent chemotherapy resistance was partially mediated by enhanced expression of DNA repair genes, BRIP, and Rad51, respectively, although it is likely that other FoxM1 targets with repair roles are also involved (Monteiro et al., 2012; Zhang et al., 2012a). Besides DNA repair, it is also possible that other FoxM1 functions contribute to chemotherapy resistance. For instance, FoxM1 is critical for checkpoint recovery upon doxorubicin or IR treatment. Following a DNA damage-induced G2 arrest, FoxM1 transcriptional activity is required to maintain the expression of pro-mitotic genes, such as cyclins or Plk1, in order to allow re-entry into the cell cycle, once the damage is repaired (Alvarez-Fernandez et al., 2010). Moreover, a recent study has proposed that FoxM1 may inhibit DNA-damage-induced apoptosis by upregulating the proapototic Bcl-2, although it has not been confirmed to be a direct FoxM1 transcriptional target (Halasi and Gartel, 2012a). These data suggest that FoxM1 might induce genotoxic resistance at different levels, through the enhancement of DNA damage repair, by favoring recovery after damage and preventing apoptosis (Figure 1).

Not only does FoxM1 control the DNA damage response, FoxM1 itself is also regulated in response to DNA damage. Stabilization of FoxM1 upon damage has been reported in several cell lines upon different DNA damaging insults, mostly at the protein level (Tan et al., 2007; Teh et al., 2010). Treatment with different DNA damaging agents, such as IR, etoposide, or UV, were reported to induce FoxM1 phosphorylation by Chk2 and stabilization at the protein level (Tan et al., 2007). On the contrary, other studies have shown that FoxM1 is repressed by p53 in response to damage (Barsotti and Prives, 2009; Pandit et al., 2009), while in other cases, no effect on FoxM1 expression was detected in response to damaging insults (Alvarez-Fernandez et al., 2010; Halasi and Gartel, 2012a). Therefore, it seems that FoxM1 regulation by the DNA damage response varies depending on the cellular context and, as such, the mechanisms controlling such regulation require further investigation.

## FoxM1 and senescence

In addition to the increase in DNA damage, MEFs derived from FoxM1 knockout mice also showed premature senescence (Wang et al., 2005). Consistent with this, FoxM1 was reported to be one of the most significantly downregulated transcription factors in human fibroblasts undergoing senescence upon activation of the p16-Rb and p53-21 pathways, and overexpression of its constitutive active form was sufficient to bypass senescence in this system (Rovillain et al., 2011). In agreement with this, FoxM1 overexpression was able to prevent oxidative stress-induced senescence in mouse fibroblasts (3T3), and this correlated with the induction of the Polycomb group protein Bmi-1, a major negative regulator of the Ink4a/Arf/Ink4b locus that encodes p19Arf as well as the cyclin-dependent kinase inhibitors p16 and p15 (Li et al., 2008). However, it has not been demonstrated that Bmi-1 is the transcriptional target of FoxM1 mediating this effect.

In a more recent study, human primary fibroblasts (IMR90) depleted of FoxM1 also exhibited a senescence-like phenotype and were more sensitive to H_2_O_2_-induced senescence, indicating again that FoxM1 could protect cells from oxidative stress (Park et al., 2009). From the molecular point of view, the same study demonstrated that FoxM1 regulates intracellular levels of ROS through transcriptional induction of anti-oxidant genes, such as catalase and MnSOD (Park et al., 2009). These previous findings of FoxM1 exhibiting senescence-suppressing activity were confirmed in a recent study using FoxM1 null MEFs challenged with the ROS inducing drug Imexon (Anders et al., 2011). In this work, FoxM1 was also identified as a critical substrate of CDK4/6 kinases that mediates senescence suppression in cancer cells through ROS regulation and the activation of genes required for the G1/S transcription (Anders et al., 2011) (Figure 1).

## Therapeutic opportunities of targeting FoxM1

During the last years, significant progress has been made in targeting FoxM1 in cancer [for a recent review see (Halasi and Gartel, 2012b)]. The above described functions of FoxM1 in DNA damage response and senescence pathways open new scenarios in which targeting FoxM1 might be of clinical benefit. Its role in DSB HR repair makes it an attractive target for combination therapies with treatments that render cells dependent on that DNA repair mechanism. That is the case for DSBs inducing agents, such as topoisomerase inhibitors, IR, or alkylators. Indeed, recent studies have already shown that targeting FoxM1 sensitizes different tumor cells to those DNA damaging treatments (Kwok et al., 2010; Halasi and Gartel, 2012a; Zhang et al., 2012a). Another interesting strategy is the possibility of combining FoxM1 inhibition with PARP inhibitors. PARP polymerases are required to repair single strand DNA breaks occurring during replication. When inhibited, unrepaired single-stranded DNA breaks result in stalled replication forks, which must be repaired by HR. Inhibiting HR, by means of FoxM1 suppression, may therefore sensitize these cells to PARP inhibitors.

The senescence suppression role of FoxM1 also offers possibilities of therapeutic intervention. That is the case of malignant melanoma, which is characterized for its chemoresistance. A recent study, already mentioned above, showed that treatment with CDK4/6 inhibitors, which inhibits FoxM1, triggered a strong senescence response in all melanoma cell lines tested but not in primary melanocytes (Anders et al., 2011). This provides an excellent therapeutic window for targeting Cdk4/6-FoxM1 signaling in order to reactivate a senescence program in cancer cells. Moreover, it is now becoming apparent that most conventional chemotherapies, which aim to induce extensive damage and apoptotic responses, are accompanied by a robust and concomitant induction of senescence (te Poele et al., 2002). Interestingly, it is also now evident that senescent cells can be cleared *in vivo* through the innate immune response (Xue et al., 2007). Therefore, combination of chemotherapy or IR with FoxM1 inhibition might improve the efficacy of those cancer treatments. Moreover, depending on the type of DNA lesion induced, FoxM1 inhibition might work at multiple levels, by blocking both DNA repair and checkpoint recovery processes, and promoting senescence.

One of the current limitations of targeting FoxM1 in cancer is its “druggability,” being a transcription factor. In the past years different compounds have been identified as FoxM1 inhibitors, such as the thiazole antibiotics Syomicin A (Radhakrishnan et al., 2006) and Thiostrepton (Bhat et al., 2009b). Surprisingly, these inhibitors do not affect the transcriptional activity of FoxM1 *per se*, but inhibit its expression at both mRNA and protein levels through an unknown mechanism (Bhat et al., 2009a). Other general proteasome inhibitors, such as bortezomib or MG132, also affected FoxM1 at the same level (Bhat et al., 2009a). In addition to this, the Balasubramanian group provided an alternative mode of action for Thiostrepton (Hegde et al., 2011). They showed that thiostrepton directly binds FoxM1, blocking its binding to the promoters of its target genes. Obviously, further studies will be required to elucidate the precise mechanism of action of these compounds, and this will hopefully help to develop more selective and specific FoxM1 inhibitors for cancer treatment.

## Closing remarks

FoxM1 is a promising and attractive target for cancer therapy. Significant progress has been made in the past years on FoxM1 function in DNA damage and senescence pathways, as well as in the possibilities of targeting FoxM1 in cancer. However, more studies are needed to further understand the precise mechanisms and transcriptional targets involved in those functions, in different cellular contexts and tumor types, and in response to different types of damage. This, together with the development of more specific inhibitors, will definitely help to define the proper therapeutic window in which targeting FoxM1 can achieve clinical benefits.

### Conflict of interest statement

The authors declare that the research was conducted in the absence of any commercial or financial relationships that could be construed as a potential conflict of interest.
